# Deep learning-based identification, classification and segmentation of infraosseous periodontal defects using a YOLOv8 neural network

**DOI:** 10.1186/s12903-026-08919-x

**Published:** 2026-06-23

**Authors:** Xukai Gu, Petra Orosz, Kristof Somodi, Xinda Li, Lei Yang, Hong Yang, Christoph Ramseier, Daniel Palkovics

**Affiliations:** 1 Department of Periodontology, Semmelweis University, Szentkirályi Street 47, Budapest, 1088 Hungary; 2 Department of Orthopedics, The First Affiliated Hospital of Harbin Medical University, Harbin, 150001 China; 3 Department of Pharmacology (State-Province Key Laboratories of Biomedicine-Pharmaceutics of China, Key Laboratory of Cardiovascular Research, Ministry of Education), College of Pharmacy, Harbin Medical University, Harbin, 150081 China; 4 Department of Periodontology, University of Bern, Freiburgstrasse 7, Bern, 3010 Switzerland

**Keywords:** Artificial intelligence, Deep learning, Dental radiology, Periapical radiograph, Periodontal defect, Image segmentation

## Abstract

**Background:**

The defect morphology determines single-tooth prognosis, surgical planning, and regenerative potential of intrabony defects. 2D radiographs are often compromised by superimposition of anatomical structures, leading to potential misinterpretation and diagnostic inaccuracies. The aim of this study was to develop YOLOv8 model to identify, classify and segment periodontal defects.

**Methods:**

A dataset of 329 periapical radiographs from the Department of Periodontology, Semmelweis University, was utilized. All radiographs were preprocessed and manually annotated. The YOLOv8 neural network was trained and mainly assessed by area under the receiver operating characteristic curve (AUC–ROC), macro-average F1-score, Intersection over Union (IoU) and Dice similarity coefficient (DSC).

**Results:**

The model achieved an AUC–ROC of 0.8078 (95% CI: 0.6633–0.9257). The macro-average F1-score was found to be 0.6559. IoU and DSC value averaged 0.6881 ± 0.2398 and 0.7789 ± 0.2664. High spatial overlap was observed for one -wall (DSC: 0.8688), three-wall (DSC: 0.8723) and four-wall (DSC: 0.8641) defects.

**Conclusions:**

The utilized YOLOv8 model demonstrated the capability to identify, classify, and segment infraosseous periodontal defects, achieving good discriminative power and moderate classification/ segmentation performance. Future work will have to focus on constructing a larger and even more refined training dataset to further enhance model performance.

**Supplementary Information:**

The online version contains supplementary material available at https://doi.org/10.1186/s12903-026-08919-x.

## Background

Periodontal defects can be classified into supraosseous defects, infraosseous defects, and furcation lesions. Infraosseous defects are primarily located at the interproximal area and can affect either a single tooth (intrabony defects) or involve both adjacent teeth (interroot defects). Intrabony defects are further categorized according to the number of residual walls, as one-, two-, three- and four wall defects. Development of different defect morphologies is influenced by various anatomical factors, such as periodontal phenotype, tooth position, root configuration [1, 2]. As the American Academy of Periodontology (AAP) Regeneration Workshop (2014) and the European Federation of Periodontology (EFP) treatment guidelines emphasized, the importance of defect-specific factors for determining single-tooth prognosis, surgical planning, and regenerative potential of intrabony defects [2–9]. Shallower- and/or wider defects with a lower number of residual walls are negatively associated with new attachment formation [3, 5, 6, 9].

Parallel long-cone intraoral radiography (IR) is the gold standard for radiographic evaluation of periodontal defect morphologies [10, 11]. While cone-beam computed tomography (CBCT) can depict periodontal defect morphology in 3D, elevated radiation doses limit their potential as a diagnostic tool in periodontology [10, 12]. On the other hand IRs provide a 2D image of 3D objects, where superimposition of anatomical structures can obscure the actual morphology of periodontal defects. Consequently, leading to potential misinterpretation and diagnostic inaccuracies [13–15].

As an emerging technology, artificial intelligence (AI) and its various subsets were proposed to circumvent the limitations of radiographic diagnostic methods. Similar to other medical and dental disciplines deep learning (DL) applications in periodontology are also mostly focused on radiographic image analysis and image processing [16]. In this context, AI processes can be integrated into the existing radiographic periodontal diagnostic workflow at two distinct stages, each fully compatible with the current diagnostic infrastructure. The first point of integration is CBCT image segmentation, enabling the acquisition of 3D virtual models of the periodontally compromised dentition, while the second target involves DL–assisted analysis of IRs.

Studies – including one published by our group—have described the use of DL-based segmentation of periodontal bone on CBCT images [17]. However, given the limited use of CBCT imaging in periodontology, the aforementioned second target for AI integration—DL-assisted analysis of IRs— might represent a clinically more feasible and scalable approach. At least until clinical guidelines change or CBCT technology develops further.

Initially, the majority of AI models in periodontology focused on the binary identification of alveolar bone loss. While these studies demonstrate high accuracy in alerting clinicians to the presence of periodontal disease [18–21], they provide limited information for treatment planning, especially for surgical decision-making. Recent advancements have shifted towards classification and segmentation tasks; however, most of these studies remain restricted to categorizing the stages of periodontitis. For example, Erturk et al. [22] utilized YOLOv8 to automatically classify periodontitis stages based on radiological bone loss, achieving an F1-score of 81.09%. While this highlights that YOLOv8 has good classification capabilities for radiological bone loss. The morphological classification of bony defects is more challenging than periodontitis staging due to the interference of anatomical superimposition and the need to capture subtle features, especially on two-dimensional radiographs. Piroonsan et al. reported binary identification performance of three-wall defects using six different DL models [23]. Area under the curve (AUC) values ranging between 0.70 and 0.75, however, these models lacked generalizability in terms of defect morphology classification. More recently a YOLOv8 DL model was trained for the classification of periodontal infrabony and furcation defects. Authors reported a moderate mean precision value of 0.592 [24], however, the model did not differentiate two- and three-wall defects. Similarly, Xia et al. [25] trained the YOLOv8 model to parameterize infraoseeous defects. However, they did not include the assessment of residual bony walls which is critical for personalized treatment planning.

Thus, the aim of the current study was to develop and evaluate the performance of a YOLOv8 DL model for the identification, classification and segmentation of one- two-, three-, four-wall as well as furcation defects.

## Materials and methods

### Study design

This retrospective single-center study collected IRs of patients who received periodontal treatment at the Department of Periodontology, Semmelweis University. A total of 329 IRs were selected for the training, validation and testing of a YOLOv8 DL model. All images were preprocessed and combined into a uniform database. Data splitting was performed at the patient level. Images were divided into a training (243 IRs), a validation (26 IRs), and a test dataset (60 IRs). The test set comprised images from patients not included in the training or validation cohorts, ensuring a fully independent evaluation.

**Fig. 1:**
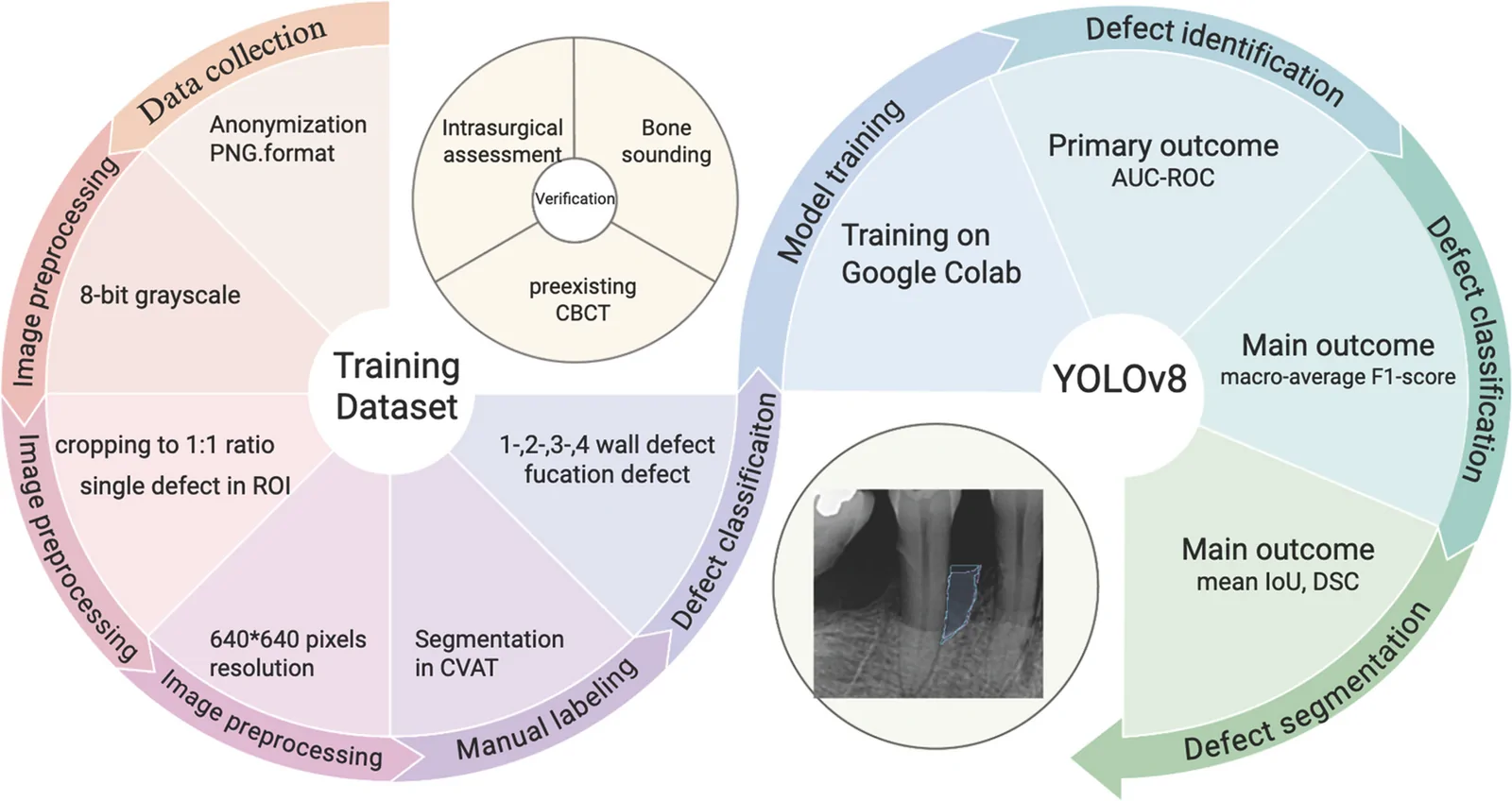
Workflow of proposed framework. Left loop: Training dataset construction. Top inset: Verification of classification. Right loop: YOLOv8 training and assessment. Bottle inset: Model’s segmentation (blue) and Manual segmentation (purple)

The study was conducted according to the Declaration of Helsinki [26], and the protocol was approved by the Regional Institutional Scientific and Research Ethics Committee (approval number SE RKEB 26/2026). The examinations followed the guidelines of Schwendicke et al. [27] and the Checklist for Artificial Intelligence in Medical Imaging (CLAIM) guidelines [28]. All images were anonymized for development of the DL model. Training was performed with GPU acceleration using an ephemeral virtual machine provided by Google Colab (https://colab.research.google.com/) (Fig. 1).

### Eligibility criteria

IRs presenting infraosseous defects and furcation lesions were included in the current study. Images were excluded if they have any of the following criteria: (i) poor image quality (e.g., severe artifacts); (ii) non-periodontal lesions (e.g., cysts, periapical lesions), (iv) bitewing radiographs, (v) orthopantomography images. IRs were derived from routine clinical practice, and no additional images were acquired specifically for the purpose of this study.

### Preparation of training database

IRs retrieved from the patient management software, were anonymized, and saved in PNG format. Image preprocessing included the following steps:

1. Conversion to 8-bit grayscale images
2. Cropping to a 1:1 aspect ratio to include a single region of interest (ROI) containing the defect
3. Resizing to a resolution of 620 × 620 pixels at 72 ppi

**Fig. 2:**
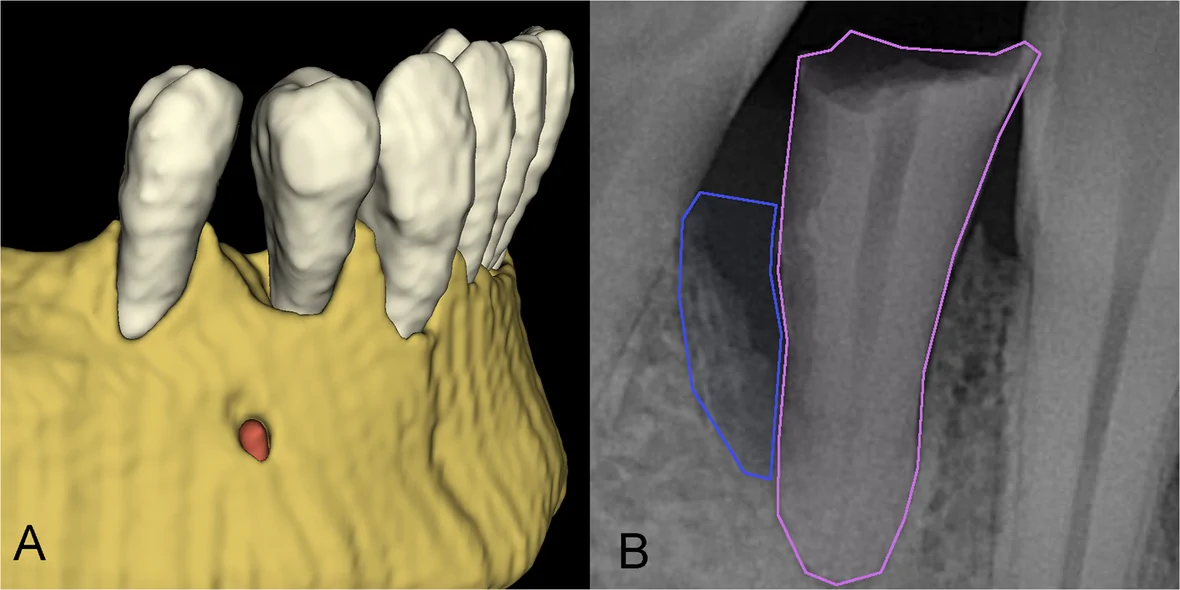
Verification of defect morphology. **A** Three-dimensional defect morphology in 3D Slicer. **B** Manual segmentation in CVAT

Infraosseous defect morphologies were assigned as follows: one-wall, two-wall, three-wall, four-wall (circumferential defects) and furcation defects. Three-dimensional defect morphology and the number of residual bone walls were confirmed by either (i) preexisting CBCT images of the patient, (ii) direct intrasurgical measurements or (iii) bone sounding measurements (Fig. 2a). All images were subsequently labeled by polygonal segmentation in an online annotation platform (CVAT, https://www.cvat.ai/) (Fig. 2b).

The distribution of defect classes across the training, validation, and test datasets is presented in Table 1.

**Table 1:** Composition of training, validation and testing datasets

	Training	Validation	Testing
Maxilla	110	10	14
Mandibula	133	16	16
Incisor	49	5	4
Canine	35	4	4
Premolar	46	6	6
Molar	113	11	16
One-wall	31	4	4
Two-wall	78	8	8
Three-wall	44	4	6
Four-wall	36	4	6
Furcation	54	6	6

### AI architecture

**Fig. 3:**
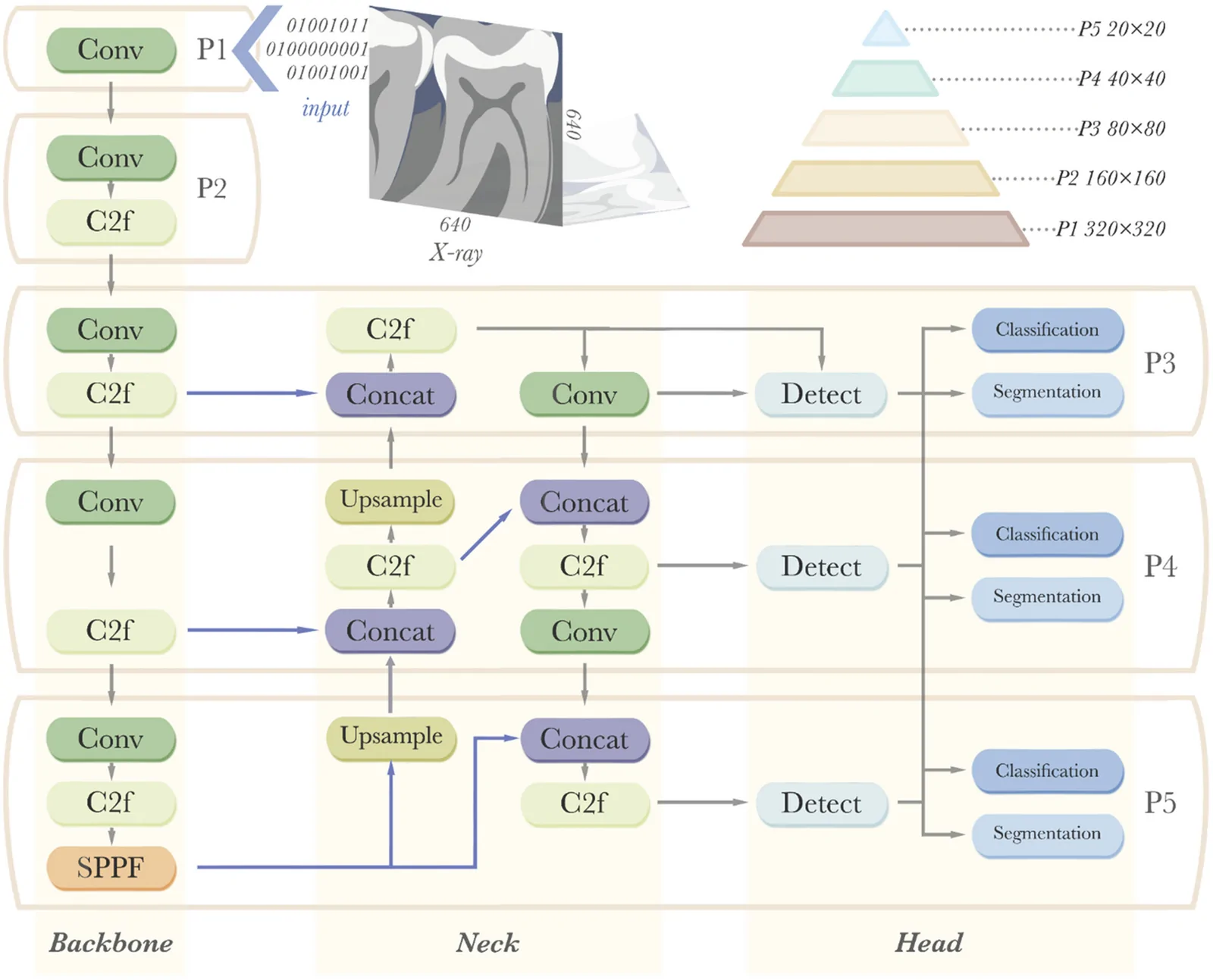
Detailed architecture of YOLOv8 neural network. Backbone: Feature extraction. Neck: Feature fusion. Head: Multi-task output. P1-5: Features map levels corresponding to different spatial resolution. Blue arrow: Interaction between superficial spatial localization features to deep semantic features

YOLOv8 (Ultralytics; https://docs.ultralytics.com/models/yolov8/) is a convolutional neural network (CNN) using an advanced cross stage partial (CSP) backbone, which enables feature splitting and cross-stage feature fusion [29]. In the present study, the YOLOv8s-seg (small-sized segmentation) variant was utilized. Compared with previous versions (YOLOv7), the C3 module is replaced by a more lightweight C2f module, allowing intermediate feature preservation. The neck architecture applies a combined feature pyramid network (FPN) and path aggregation network (PAN) design, facilitating interaction between deep semantic features and superficial spatial localization features. The Ultralytics detection head employs an anchor-free decoupled design. Which facilitates improved stability and enhanced sensitivity to small and irregularly shaped objects and features (Fig. 3). Furthermore, when comparing with two-stage CNNs (e.g. Mask R-CNN) or segmentation network (e.g. U-Net), YOLOv8’s single-stage pipeline unifies defect identification, multi-class classification, and segmentation into a highly efficient process, making it far more suitable for real-time clinical deployment.

Ultralytics provides models suitable for different tasks (identification, classification, segmentation, pose, oriented detection) and includes different sizes: nano, small, medium, large, and extra-large. We utilized the YOLOv8s-seg model to complete identification, classification, and segmentation.

### Model training

**Fig. 4:**
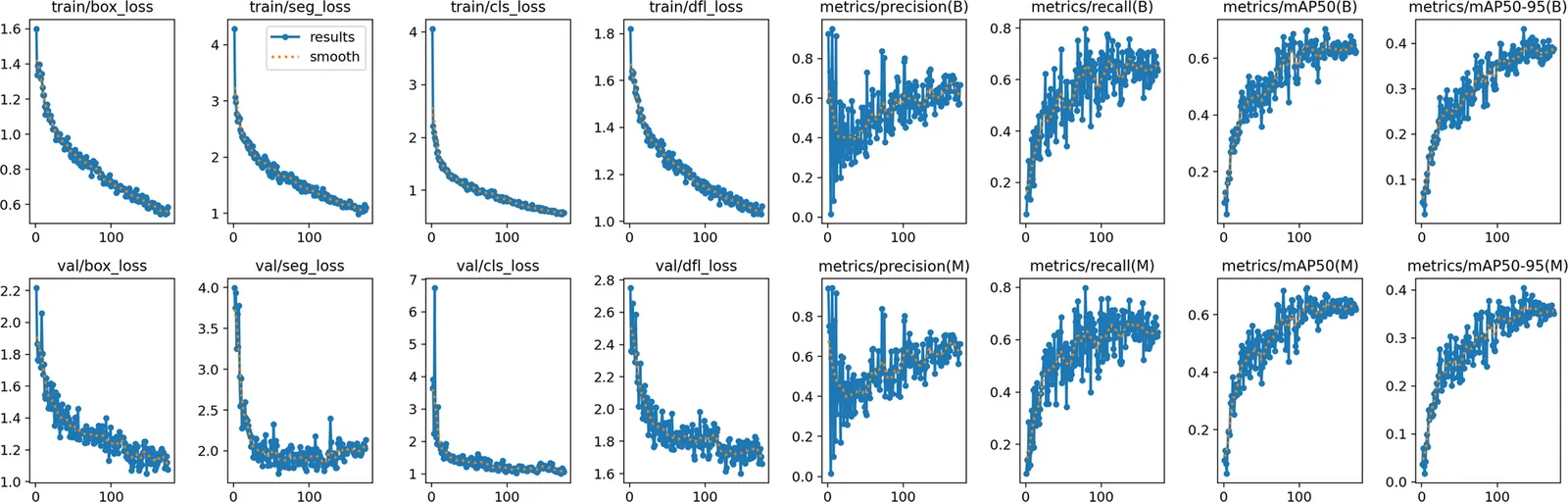
Training and validation curves of the YOLOv8 segmentation model. The upper row shows training loss components (box, segmentation, classification, and distribution focal loss) and performance metrics over epochs. The lower row presents the corresponding validation losses and metrics

Training was conducted with GPU acceleration using an NVIDIA Tesla T4 GPU (Santa Clara, CA, USA) on the Google Colab platform (Mountain View, CA, USA). The model was trained for 175 epochs. Convergence was assessed by monitoring the training and validation losses, which decreased and stabilized in the final epochs, accompanied by plateauing (Fig. 4). Based on GPU capacity a batch size of 4 was selected based on GPU memory and to maintain stable training. Optimization was performed using the default Ultralytics YOLOv8 configuration, which automatically selected the AdamW optimizer with an initial learning rate of approximately 0.001. The model was trained using the standard loss functions implemented in YOLOv8, including bounding box regression loss, classification loss, and segmentation mask loss.

Data augmentation was applied using the default Ultralytics YOLOv8 pipeline, including random horizontal flipping, scaling, translation, and mosaic augmentation. Additional intensity-based transformations included color space adjustments and Albumentation-based preprocessing (blur, grayscale conversion). Vertical flipping was not applied.

### Outcome variables

#### Identification performance

Primary outcome of the study was to determine the model’s capability to identify the presence of infraosseous defects using the area under the receiver operating characteristic curve (AUC–ROC) [30]. To evaluate binary defect identification performance a complementary set of IRs without defects was introduced as a negative control. IRs were selected from an independent pool and paired with the defect-positive radiographs in a 1:1 ratio for AUC–ROC computation. A 95% confidence interval (CI) with non-parametric bootstrapping was used for AUC–ROC calculation. Additionally, to AUC-ROC value the model’s identification performance was also assessed with the following metrics: accuracy, sensitivity, specificity, precision/positive predictive value (PPV), negative predictive value (NPV). Metrics were calculated from true positive (TP), true negative (TN), false positive (FP) and false negative (FN) values using the formulas shown below.

#### Classification performance

The current YOLOv8 model was trained to classify infraosseous and furcation defects according to the previously established categories.

The F1 score was used as the primary index to assess the model’s accuracy in defect classification [31]. In addition, accuracy, sensitivity, specificity, PPV, NPV was also evaluated as secondary indices. A confusion matrix will be used to calculate the above metrics:

- $$F1 score=\frac{2TP}{2TP+FP+FN}$$
- $$Accuracy=\frac{TP+TN}{TP+TN+FP+FN}$$
- $$Sensitivity=\frac{TP}{TP+FN}$$
- $$Specificity=\frac{TN}{TN+FP}$$
- $$PPV=\frac{TP}{TP+FP}$$
- $$NPV=\frac{TN}{TN+FN}$$

#### Segmentation performance

Results of the DL segmentation were compared to ground truth manual segmentations performed by a trained professional. The performance of the DL model was assessed by quantifying the spatial overlap of pixels labeled by the YOLOv8 model and the investigator using the Intersection over Union (IoU) and the Dice similarity coefficient (DSC) metrics [32].

- $$IoU=\frac{TP}{TP+FP+FN}$$
- $$DSC=\frac{2TP}{2TP+FP+FN}$$

### Statistical analysis

Model performance for defect identification was evaluated using AUC-ROC with a bootstrapped 95% CIs. For classification tasks, performance was assessed using accuracy, sensitivity, specificity, PPV, NPV, and F1-score. Class-specific metrics were calculated using a one-vs-rest approach. Macro-average values were computed as the unweighted means of class-specific metrics. CIs for performance metrics were calculated using the Wilson score method. Confidence intervals for the macro-average F1-score were estimated using bootstrap resampling (1000 iterations). Segmentation performance was evaluated using IoU and DSC, and results are reported as mean ± standard deviation. Statistical analyses were performed using Stata (version 18; StataCorp, College Station, TX, USA).

## Results

### Identification performance

**Fig. 5:**
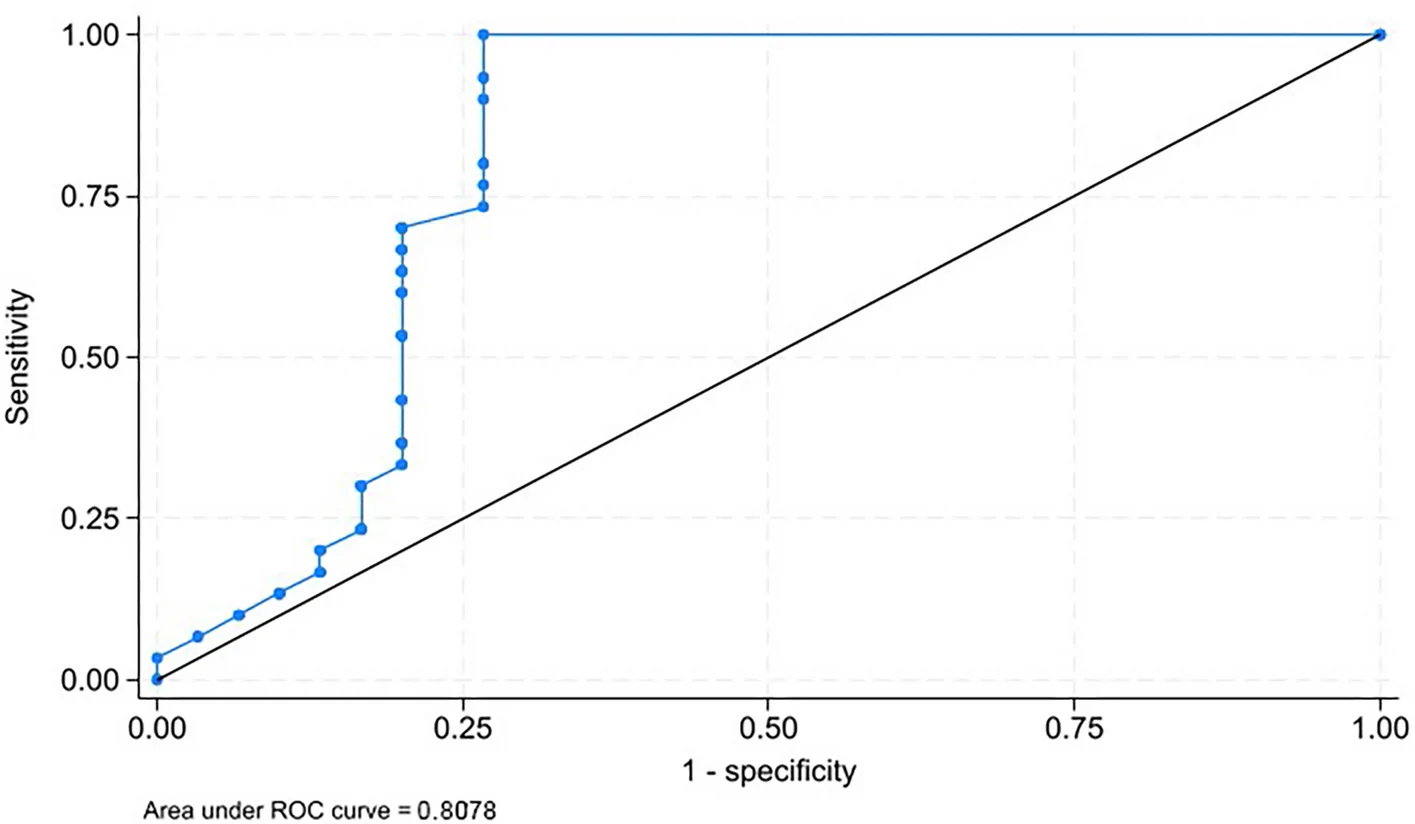
The area under the receiver operating characteristic (AUC-ROC) curve. X-axis: Sensitivity. Y-axis: 1-Specificity. Area under ROC curve: 0.8078

The goal of defect identification was to assess whether the YOLOv8 model could distinguish infraosseous defects from a healthy periodontium. On the testing dataset containing also the negative control IRs the model achieved an AUC of 0.8078 (95% conf.int. 0.6633–0.9257) for the identification of infraosseous defects. This value indicated a good performance in the model’s identification capability. The AUC-ROC is visualized in Fig. 5. The model showed an average accuracy of 0.8667, sensitivity of 1.000, a relatively lower specificity of 0.7333, PPV of 0.7895 and a NPV of 1.0000 respectively.

### Classification performance

The macro-average F1-score was calculated to determine the model’s overall performance in the classification of infraossues defects, which was found to be 0.6559. When the F1 score was analyzed on a per-class basis, the model’s class-specific F1 scores were as follows: 0.5714 (1-wall), 0.6667 (2-wall), 0.7273 (3-wall), 0.7143 (4-wall), and 0.6000 (furcation).

**Fig. 6:**
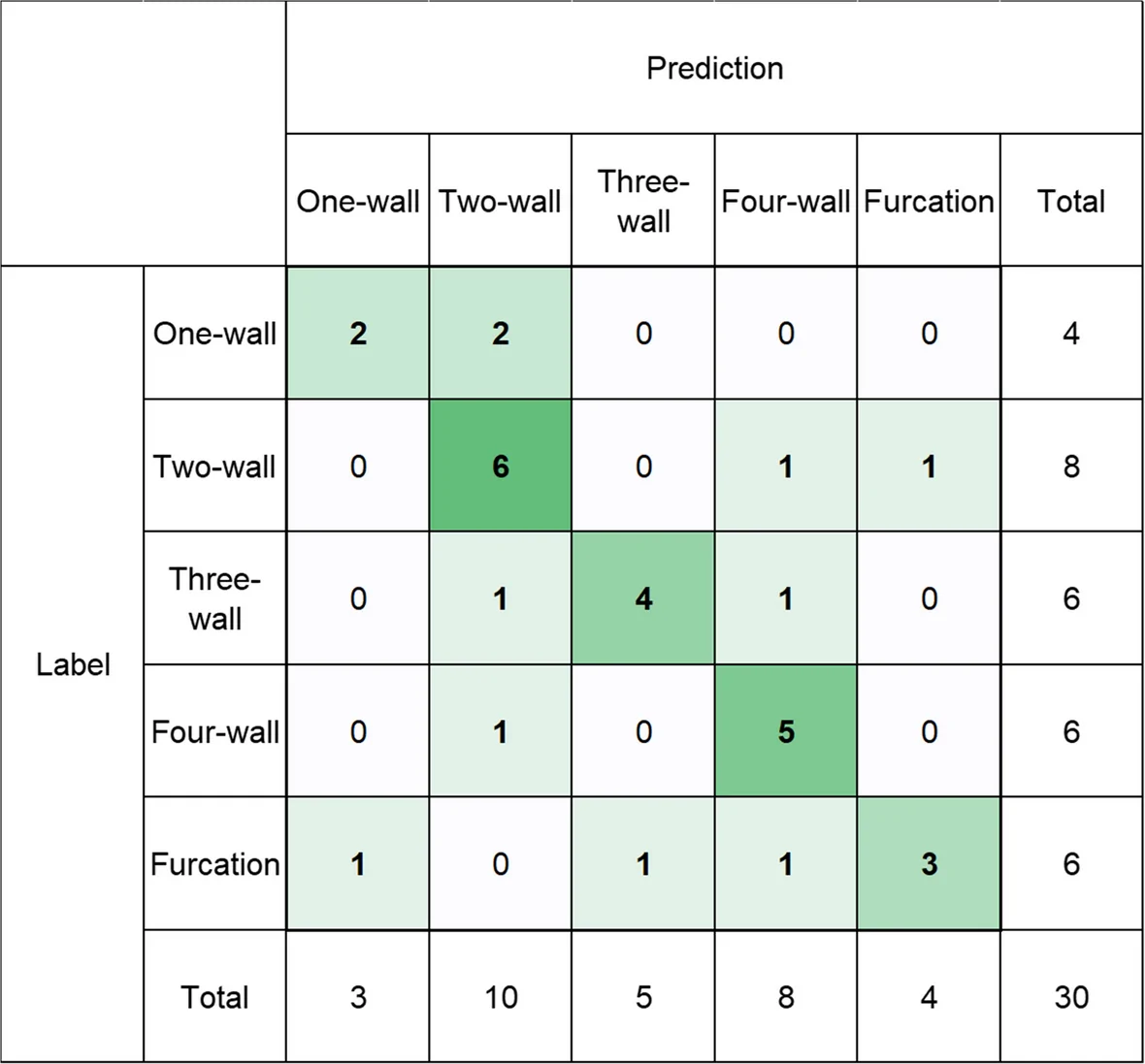
Confusion matrix illustrating classification performance across defect categories

Similarly to the identification performance, overall as well as class-specific accuracy, sensitivity, specificity, PPV, NPV scores were calculated. Values are shown in Table 2. Confusion matrix is shown in Fig. 6.

**Table 2:** Class-specific metrics of classification performance

Defect class	F1-score	Accuracy (95% CI^a^)	Sensitivity (95% CI)	Specificity (95% CI)	PPV^b^ (95% CI)	NPV^c^ (95% CI)
One-wall	0.5714	0.9000 (0.7438 – 0.9654)	0.5000 (0.1500 – 0.8499)	0.9615 (0.8111 – 0.9932)	0.6667 (0.2077 – 0.9385)	0.9259 (0.7663 – 0.9794)
Two-wall	0.6667	0.8000 (0.6269 – 0.9049)	0.7500 (0.4093 – 0.9285)	0.8181 (0.6148 – 0.9269)	0.6000 (0.3127 – 0.8318)	0.9000 (0.6989 – 0.9721)
Three-wall	0.7273	0.9000 (0.7437 – 0.9654)	0.6667 (0.2999 – 0.9032)	0.9583 (0.7976 – 0.9926)	0.8000 (0.3755 – 0.9637)	0.9200 (0.7503 – 0.9777)
Four-wall	0.7143	0.8667 (0.7032 – 0.9469)	0.8333 (0.4365 – 0.9699)	0.8750 (0.6899 – 0.9566)	0.6250 (0.3057 – 0.8632)	0.9545 0.7820 – 0.9919)
Furcation	0.6000	0.8667 (0.7032 – 0.9469)	0.5000 (0.1877 – 0.8124)	0.9583 (0.7976 – 0.9926)	0.7500 (0.3006 – 0.9544)	0.8846 (0.7102 – 0.9599)
Macro-average	0.6559 (95% BCI^d^: 0.4608—0.8511)	na	na	na	na	na

^a^Confidence Interval

^b^Positive predictive value

^c^Negative predictive value

^d^Bootstrapped confidence interval

### Segmentation performance

**Fig. 7:**
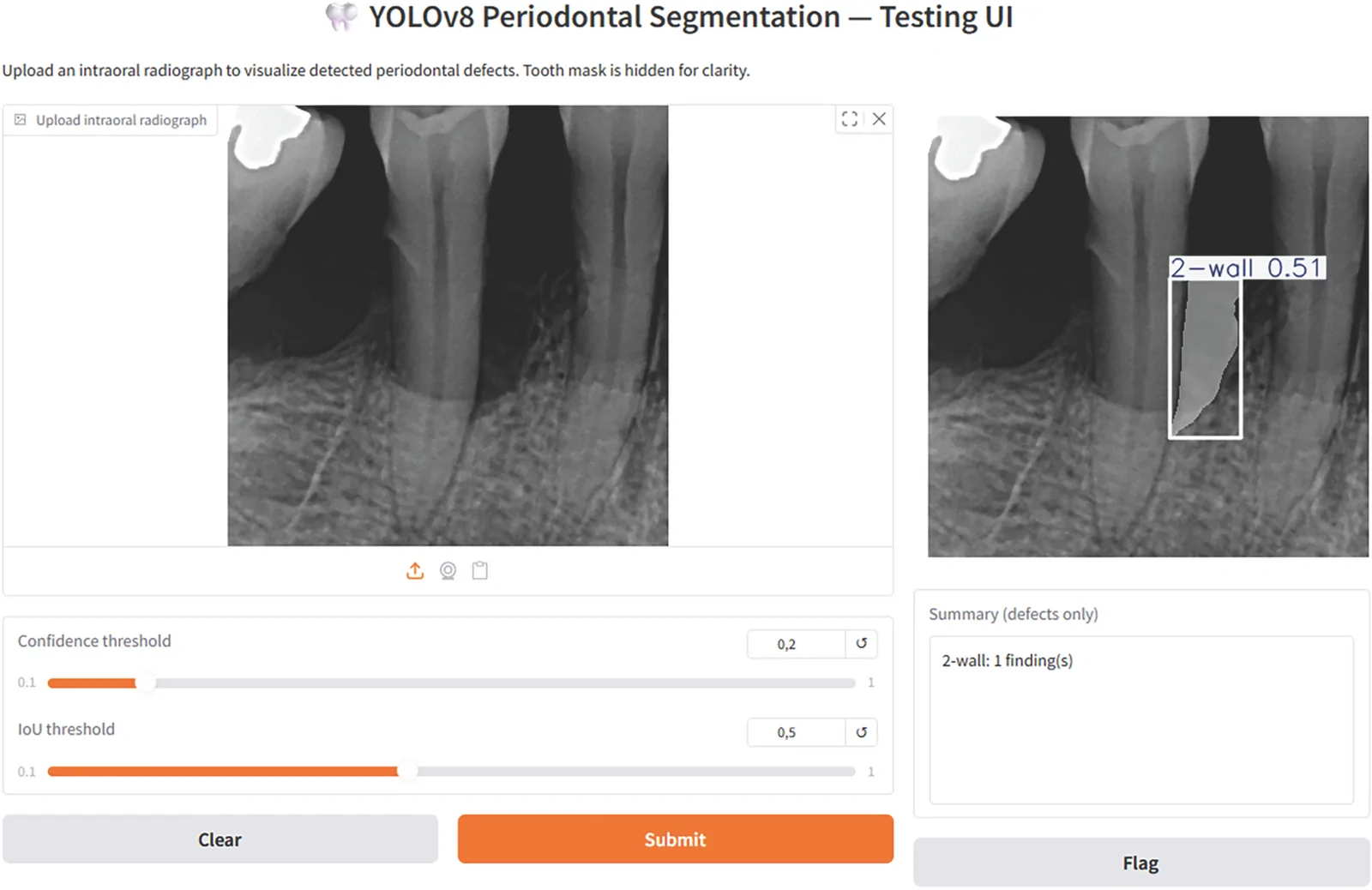
Testing of trained YOLOv8 model. The trained YOLOv8 model categorized defects based on the remaining number of bone walls, and results was accompanied by a probability score

**Fig. 8:**
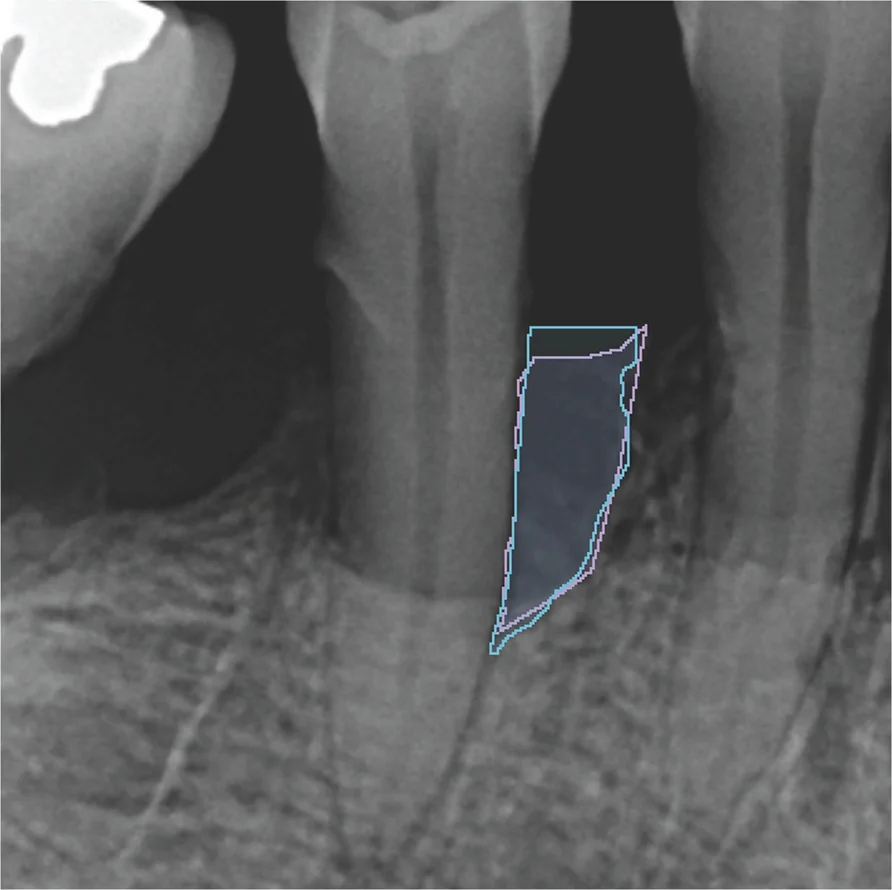
Spatial overlapping of model’s and ground truth segmentation. Model’s segmentation (blue) and ground truth manual segmentation (purple)

The mean IoU and DSC value between YOLOv8 model’s segmentation and the ground truth segmentation was 0.6881 ± 0.2398 and 0.7789 ± 0.2664, indicating a moderate similarity (Figs. 7, 8). Mean class-specific IoU and DSC values were 0.7696 ± 0.0621 and 0.8688 ± 0.0397 (one-wall); 0.6646 ± 0.2698 and 0.7553 ± 0.3056 (two-wall); 0.7750 ± 0.0581 and 0.8723 ± 0.0365 (three-wall); 0.7624 ± 0.0622 and 0.8641 ± 0.0390 (four-wall); 0.5038 ± 0.3994 and 0.57186 ± 0.4461 (furcation). Individual IoU and DSC values are shown in Table 3.

**Table 3:** Metrics of segmentation performance

Reference number	IoU^a^	DSC^b^
1	0.7995	0.8886
2	0.7506	0.8575
3	0.7248	0.8405
4	0.8168	0.8991
5	0.8290	0.9065
6	0.7769	0.8745
7	0.7078	0.8289
8	0.0000	0.0000
9	0.7696	0.8698
10	0.7273	0.8421
11	0.7247	0.8404
12	0.7686	0.8692
13	0.7191	0.8366
14	0.7153	0.8340
15	0.8103	0.8952
16	0.7517	0.8583
17	0.8656	0.9280
18	0.7883	0.8816
19	0.8631	0.9265
20	0.8166	0.8991
21	0.7268	0.8418
22	0.7303	0.8442
23	0.7274	0.8422
24	0.7103	0.8306
25	0.0000	0.0000
26	0.7431	0.8526
27	0.7202	0.8373
28	0.0000	0.0000
29	0.6503	0.7881
30	0.9092	0.9525
Mean ± st. dev^c^	0.6881 ± 0.2398	0.7789 ± 0.2664

^a^Intersection over union

^b^Dice similarity coefficient

^c^Standard deviation

### Failure case analysis

**Fig. 9:**
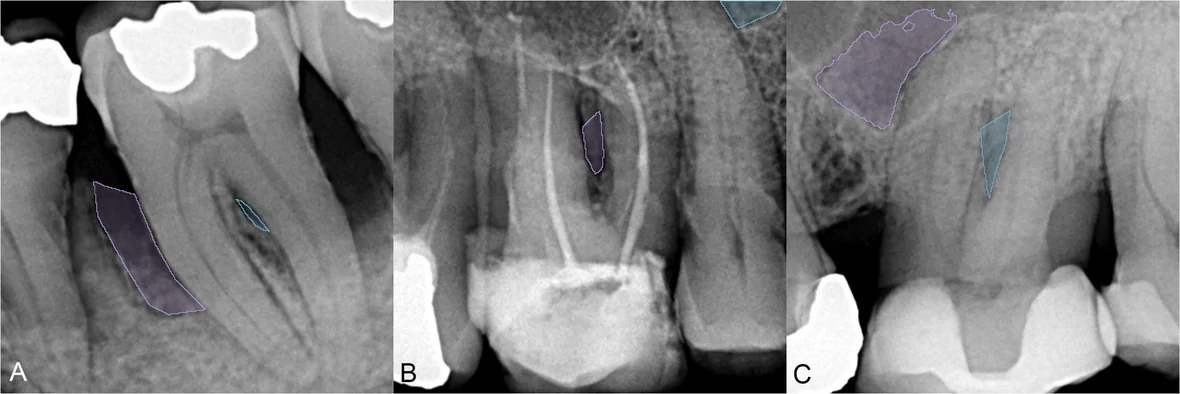
Complete misclassification cases. **A** Lower molar case (#8) in which the model misclassified a two-wall defect as a furcation lesion, likely due to increased radiolucency between the roots. **B**-**C** Maxillary molar cases (#25 and #28) where the model incorrectly segmented the radiolucent region of the maxillary sinus, classifying it as a three-wall and a two-wall defect, respectively. In these cases, no spatial overlap was observed between the model prediction and the ground truth segmentation

In three cases the model completely misclassified the defects, as a result the model’s segmentations showed no spatial overlap with the ground truth. All complete failures occurred in images involving molars. In one case with a lower molar (case #8), the model misclassified the defect as a furcation lesion instead of the reference two-wall defect, likely due to the increased radiolucency between the roots (Fig. 9a). In the remaining two cases, the model incorrectly segmented the radiolucent region of the maxillary sinus, classifying it as a three-wall defect (case #25) in one instance and as a two-wall defect (case #28) in the other (Fig. 9b, c).

## Discussion

This study evaluated the performance of a YOLOv8 model trained to aid the diagnosis of periodontal defects on IRs. By achieving moderate to acceptable results in automated identification, classification and segmentation the current study supports the feasibility of this method. Although these initial results are comparable to those reported in the literature by similar approaches, drawbacks of the current study must be noted.

The greatest hinderance of the current method is the relatively small training dataset (*n =* 243), and thus the relatively low instances of each individual defect category. This reduces the model’s robustness, making the network more susceptible to overfitting. Furthermore, it also limits the model’s generalizability, as the training dataset may not include all morphological variations of infraosseous defects visible in a broader population. Another limitation of the present study is that model performance was evaluated using a single internal test set. Although strict patient-level separation was applied, this approach does not fully reflect variability across different datasets. The absence of cross-validation and external validation limits the strength of conclusions regarding model generalizability. These limitations likely contributed to the moderate performance of the model and should be addressed in future large-scale, multi-center studies with external validation cohorts to enable a more robust assessment. It is also important to acknowledge the use of a relatively simple single stage DL architecture. While the YOLOv8 model is powerful all-in-one tool for object detection, classification and segmentation, the overall performance might be improved with a more elaborate multi-stage design.

One of the advantages of current method, that it is not restricted only to the binary identification of periodontal defects or specific sub-types [23, 33, 34]. Despite the limited training dataset and relatively simple DL architecture design, the performance of the current model is generally comparable to those found in the literature. Accordingly, previously reported AUC values for the identification of furcation defects was 0.868, whereas for vertical defects was only 0.733 [34]. Even though in the current evaluation there was no separate AUC value calculated for each category, the AUC-ROC value of 0.8078 falls in between the same range.

There are only a couple of articles reporting results of infraosseous defect classification [24, 25]. In this regard the current model achieved comparable and even slightly better results (mean PPV = 0.6883) across more classes than those reported in the paper of Valente et al. [24] (mean precisio*n =* 0.529). As both studies utilized the same YOLOv8 architecture, this difference might stem from the difference in database construction and image annotation. Additionally, the method of Valente et al. did not differentiate between two- and three-wall defects, even though there is a significant difference in the two in terms of treatment strategy and prognosis. Xia et al. [25] utilized a multi-stage DL model combining the nnU-net and YOLOv8 architectures to parametrize bone defects based on the width, depth and radiographic angle. Although these parameters are relevant for prognostic assessment, the literature consistently identifies the number of remaining bony walls as having greater predictive value for treatment outcomes. Since Xia et al. have utilized a different methodology, results cannot be directly compared to the current paper. Another strength of the present approach was the verification of three-dimensional defect morphology using CBCT imaging and/or intraoperative measurements. Consequently, the training dataset incorporated morphological information beyond what was directly visible on the IRs, thereby enhancing the biological validity of the ground truth annotations.

From a clinical standpoint, precise assessment of defect morphology is a key determinant in selecting the appropriate surgical approach and regenerative strategy. The number of residual bony walls plays a critical role in guiding flap design, determining the indication for regenerative procedures, and predicting regenerative potential. In this context, DL-assisted classification may enhance preoperative planning, and contribute to more individualized and predictable treatment decisions.

However, the clinical implications of misclassification should not be underestimated. Incorrect defect classification may result in the selection of inappropriate flap techniques or regenerative approaches, necessitating intraoperative modifications of the surgical plan. Such adjustments may prolong surgical time, increase technical complexity, and potentially compromise regenerative outcomes. Ultimately, these limitations highlight the importance of further improving model accuracy and robustness before routine clinical implementation.

Based on the strengths and limitations of the current evaluation, several points for improvement can be identified. These include expanding the size of the training dataset to enhance model robustness, incorporating external validation data to improve generalizability, and directly integrating three-dimensional data into the training pipeline. Further methodological refinement could be achieved by combining multiple DL architectures. In addition, refining the database structure through the integration of clinical probing parameters may further improve the clinical relevance and predictive performance of the model.

## Conclusion

The utilized YOLOv8 model demonstrated the capability to identify, classify, and segment infraosseous periodontal defects, achieving good discriminative power (AUC: 0.8078) and moderate classification and segmentation performance. Results achieved with even this limited training dataset were comparable to those found in the literature, showing the importance of structured data. Despite these encouraging results, the model’s performance in this current feasibility experiment was constrained by the scale of the dataset. Future work will have to focus on constructing a larger and even more refined training dataset to further enhance model performance.

## Supplementary Information

Supplementary Material 1

## Data Availability

All data generated or analyzed during this study are included in this published article.

## Abbreviations

AAP: American Academy of Periodontology
AI: Artificial intelligence
AUC-ROC: Area under the receiver operating characteristic curve
CBCT: Cone-beam computed tomography
CLAIM: Checklist for Artificial Intelligence in Medical Imaging
CNN: Convolutional neural network
CSP: Cross stage partial
DL: Deep learning
DSC: Dice similarity coefficient
EFP: European Federation of Periodontology
FPN: Feature pyramid network
GPU: Graphics processing unit
IoU: Intersection over union
IR: Intraoral radiography
NPV: Negative predictive value
PAN: Path aggregation network
PPV: Positive predictive value
ROI: Region of interest
RBL: Radiographic bone level
